# Characterization of *Congregicoccus parvus* gen. nov., sp. nov., a novel slow-growing ultramicrobacterium of the phylum *Verrucomicrobiota*

**DOI:** 10.1371/journal.pone.0326734

**Published:** 2025-06-25

**Authors:** Md. Samiul Islam, Kyosuke Yamamoto, Naoki Morita, Wataru Kitagawa, Isao Yumoto, Souichiro Kato, Ryosuke Nakai, Kensuke Igarashi

**Affiliations:** 1 Graduate School of Agriculture, Hokkaido University, Sapporo, Hokkaido, Japan; 2 Bioproduction Research Institute, National Institute of Advanced Industrial Science and Technology (AIST), Sapporo, Hokkaido, Japan; Cardiff's Metropolitan University: Cardiff Metropolitan University, UNITED KINGDOM OF GREAT BRITAIN AND NORTHERN IRELAND

## Abstract

The bacterial phylum *Verrucomicrobiota* is a typical representative of challenging-to-culture lineages. Here, we isolated a novel slow-growing *Verrucomicrobiota* bacterium, designated ASA1^T^, from garden soil. Phylogenetic analyses suggested that strain ASA1^T^ was affiliated with a novel candidate in the family *Alterococcaceae* of the class *Opitutia* (previously *Opitutae*), exhibiting low 16S rRNA gene sequence identities (<93%) and average nucleotide identity values (<77%) to the closest type strain. Furthermore, average amino acid identity analysis (AAI) showed that ASA1^T^ shares <54% AAI identity with *Opitutia* representatives, well below the 60–65% genus threshold, supporting its classification as a novel genus. In addition to its genomic characteristics, ASA1^T^ exhibits distinctive morphological features, including an ultramicrobacterial cell size (0.05–0.07 μm^3^) and cluster-like aggregation (>10 µm). Strain was Gram-negative, aerobic, and motile, with menaquinone-7 as its primary respiratory quinone and a fatty acid profile dominated by anteiso-C_15:0_ (53%) and C_16:0_ (15%). Based on these results, we propose that strain ASA1^T^ represents a novel genus and species named *Congregicoccus parvus* gen. nov., sp. nov. (JCM 36569^T^ = NCIMB 15508^T^) of the *Alterococcaceae* family. Habitability analysis suggested that ASA1^T^ relatives were distributed in the soil and other environments, such as freshwater and activated sludge. Additional genome analysis of strain ASA1^T^ revealed six unique biosynthetic gene clusters with no related clusters identified via antiSMASH, suggesting a potential source of novel bioactive compounds for future research.

## Introduction

Microorganisms, especially bacteria and archaea, are ubiquitous and diverse in natural environments [1]. However, despite their ubiquity, our understanding of microbial function remains largely unknown due to culture challenges. Recent advances in culture-independent methods, however, have unveiled hidden microbial diversity, including bacteria that are difficult to culture or slow-growing [2]. Culturing uncultured bacteria holds significance in various scientific domains, offering insights into metabolic capabilities, ecological roles, and potential biotechnological applications [3]. Furthermore, environmental bacteria play pivotal roles in maintaining ecosystem stability [4], nutrient cycling [5], and soil health [6], making their isolation and characterization imperative for understanding ecosystem dynamics and fostering environmental management.

The phylum *Verrucomicrobiota* is a typical representative of difficult-to-culture bacterial lineages [7,8]. This phylum presently comprises only 82 type strains (based on Bac*Dive* data [https://bacdive.dsmz.de/]; accessed on May 8, 2025) [9]. A series of 16S rRNA gene-based PCR surveys have shown that *Verrucomicrobiota* members are dominant (approx. 35–50%) in grassland and coniferous forest soils [7]. In particular, members of the class *Opitutia* (previously *Opitutae*) [10] show a wide distribution in paddy soils and rhizospheres, highlighting their plant-microbe interaction [7] and very small cell sizes [11,12]. Certain strains within this class have demonstrated the ability to metabolize biopolymers, such as lignin, xylan, and starch, as their sole carbon sources [13]. Additionally, some members possess photoheterotrophic metabolisms [14], while others harbor *nif* genes involved in nitrogen fixation [15].

In this study, we describe our successful isolation and characterization of a novel slow-growing *Opitutia* strain, ASA1^T^. We also characterized this strain, focusing on physiological, chemotaxonomic, phylogenetic, and phylogenomic aspects. Finally, we propose that strain ASA1^T^ represents a novel genus and species and named it *Congregicoccus parvus* gen. nov., sp. nov., which belongs to the family *Alterococcaceae* of the class *Opitutia*.

## Materials and methods

### Isolation and growth characterization on different media

The microbial specimen was collected from a depth of 5–15 cm beneath the surface of garden soil located at the National Institute of Advanced Industrial Science and Technology (AIST) Hokkaido Center (43.0201° N, 141.4182° E). The procedure for isolating strain ASA1^T^ adhered to methods outlined previously [16]. Briefly, soil samples were suspended in sterilized saline (0.9% NaCl) and subjected to a 10-fold dilution series. Aliquots (100 μL) from each dilution were streaked onto a 100 × diluted PYG (peptone, yeast extract, and glucose; phosphate and agar autoclaved separately) medium (maintained at pH 8.4) and then incubated at 30°C in the dark [16]. Following a 14-d incubation period, small colonies were chosen and transferred to fresh R2A plates (DAIGO; Nihon Pharmaceutical, Tokyo). This purification process was repeated at least three times to ensure consistent colony purity. Additionally, to optimize the growth of the ASA1^T^ strain, four additional media were employed: nutrient agar (NA), Luria-Bertani (LB), diluted PYG (1/10 and 1/100 dilutions), diluted R2A (1/10 and 1/100 dilutions), and tryptic soy agar (TSA). Sampling permission for the soil used in this study was obtained from the AIST, Hokkaido, Japan.

### Phylogenetic analysis based on 16S rRNA gene sequences

The bacterial genomic DNA was extracted using the phenol-chloroform method, with minor modifications [17]. The 16S rRNA gene sequence was amplified using two universal primers, 27F (5′- AGAGTTTGATCCTGGCTCAG-3′) and 1492R (5′-GGTTACCTTGTTACGACTT-3′) [18]. An Ex-Taq DNA polymerase kit (TaKaRa) was used to perform PCR on single colonies for identification and purity screening, with one reaction comprising 50 μL. Using 1% agarose gel electrophoresis, the expected 1.5-kb PCR products were detected and sequenced using an Applied Biosystems BigDye Terminator v3.1 Cycle Sequencing Kit (Thermo Fisher Scientific) with the sequencing primer 907R (5′-CCGYCAATTCMTTTRAGTTT-3′) and an Applied Biosystems 3500xL Genetic Analyzer (Thermo Fisher Scientific). The obtained partial sequences were compared with known sequences in the EzBioCloud database (https://www.ezbiocloud.net/) using a BLAST search and pairwise sequence alignment [19–21]. The novel candidate strain identified in this study, called ASA1^T^, was employed for subsequent analyses. The complete 16S rRNA gene sequence of ASA1^T^ was obtained from a genome sequence in a previous study (DDBJ/ENA/GenBank accession number AP028972.1) [22]. The sequence was subjected to a BLASTn search [19] against the NCBI nt/nr database (accessed on September 9, 2024) to identify the closest type strains, taxonomically undescribed strains, and uncultivated clones/phylotypes. All sequences were aligned using the MEGA11 [23] multiple alignment output by the CLUSTAL method [24]. Evolutionary distances were estimated utilizing the Kimura two-parameter model [25]. Phylogenetic trees were constructed using the neighbor-joining (NJ) [26] and maximum-likelihood (ML) methods [27], with 1,000 bootstrap replicates for all sequences. The complete genome sequence of strain ASA1^T^ was determined using the PacBio Sequel IIe (SMRT) sequencing platform, following the manufacturer’s instructions. The detailed procedure and sequencing conditions were performed as described in our previous study [22]. Based on our sequencing workflow, high-quality HiFi long reads were generated. The sequencing achieved a 51 × coverage, ensuring a high-quality genome assembly. The final assembly resulted in a single circular chromosome with no additional contigs, confirming the completeness of the genome. To create a graphical representation of the ASA1 genome map, the CGView (http://cgview.ca/) tools [28] in the KBase database (https://www.kbase.us/) [29] was used (S1 Fig in Appendix S1). In addition, the BV-BRC (https://www.bv-brc.org/) tool [30] was utilized to generate a phylogenomic tree (the genome data is only available on this platform; accessed on September 9, 2024) within the class *Opitutia*, while GTDB-Tk v2.3.2 was employed to create another tree encompassing thousands of metagenome-assembled genomes (MAGs) and other related classes obtained directly from the phylum *Verrucomicrobiota* [31]. Both trees were visualized using the interactive Tree of Life (iTOL) online [32]. The online platform KBase provides average nucleotide identity (ANI) analysis. To further refine the taxonomic placement of strain ASA1^T^, average amino acid identity (AAI) was calculated against fully sequenced *Opitutia* class representatives using the Enve-omics AAI Calculator (https://enveomics.scigap.org/), a widely used tool for genome-based taxonomy [33].

### Morphological, physiological, and biochemical characterization

For regular maintenance, the ASA1^T^ strain was cultivated on solid R2A media. In the case of liquid cultures, the cells were grown with agitation (at 180 rpm) in 50-mL sterile conical tubes with 15 mL of R2B (identical to R2A, but without agar). Unless specified otherwise, all incubations were conducted at 30°C at a pH of 8.4. Cell morphology assessment and DAPI (4’,6-diamidino-2-phenylindole) staining analysis were performed using an Axio Imager microscope (Carl Zeiss). For scanning electron microscopy (SEM) analysis, cells from exponentially growing cultures (7 d of incubation) in R2B medium were fixed with a mixture of 4% paraformaldehyde (PFA) and glutaraldehyde (GA) and then observed using a JSM-7500F SEM (JEOL Ltd., Tokyo, Japan). The detailed procedures for the SEM observations are provided in the supplementary S1 method (Appendix S1). Gram staining was also used to stain ASA1^T^ strain cells according to the manufacturer’s instructions (Sigma-Aldrich, St. Louis, MO, USA). Gram properties were also determined by the reaction of the bacterial strain with a 3% KOH solution [34]. Cell volume was calculated as described previously [35]. The motility test was performed using a soft agar medium [36]. Catalase activity was examined using 3% (v/v) H_2_O_2_, while oxidase activity was tested using an oxidase reagent (Sigma-Aldrich, St. Louis, MO, USA). To evaluate the range of temperature for growth, isolate ASA1^T^ was grown on R2A at a range of temperatures (15, 20, 25, 30, 35, 37, 40, and 45°C). The growth of the ASA1^T^ strain at different pH levels (5.0, 7.0, 8.4, 9.0, 11.0) and NaCl concentrations (0, 0.5, 1.0, 1.5, 2.0, 2.5, 3.0, 4.0, 5.0, [%, w/v]) was also assessed on R2A medium. Physiological and biochemical characteristics, along with enzyme activities, were assessed using API 20 NE and API ZYM test strips (bioMérieux; Marcy-l’Étoile, France). The utilization of different substrates was examined by cultivating 50-mL culture tubes with 1% (v/v) of an exponential-phase culture (7 d) cultivated in R2B medium. These tubes were subsequently incubated at 30°C. The filter-sterilized substrates were added so that the final concentration was 10 mM, except for starch, which was added at a final concentration of 0.1%. The test substrates included D-glucose, D-fructose, D-galactose, D-mannose, sucrose, D-ribose, D-xylose, L-arabinose, D-mannitol, D-sorbitol, D-maltose, D-cellobiose, and starch. Cell growth was measured with a spectrophotometer set to 600 nm.

### Potential distribution and habitability analysis

To assess the potential distribution and habitability of strain ASA1^T^ and its closely related strains, we utilized the integrated microbial next generation sequencing (IMNGS) platform (https://www.imngs.org/) [37], which enables a comprehensive exploration via a database search utilizing metagenome-derived 16S rRNA gene amplicon datasets. In this analysis, the 16S rRNA gene sequence of strain ASA1^T^ served as a query sequence. IMNGS was then conducted at a 97% sequence similarity threshold. Concurrently, we investigated habitat preferences using ProkAtlas, an extensive database (http://prokatlas.bs.s.u-tokyo.ac.jp/) with over 360,000 16S rRNA gene sequences categorized by one environmental type [38]. During alignment with the IMNGS, we employed the 16S rRNA gene sequence of ASA1^T^ as the query for ProkAtlas, searching for 97% similarity thresholds.

### Chemotaxonomic profiling

The chemotaxonomic characteristics of strain ASA1^T^ were assessed by analyzing its cellular fatty acid composition and identifying its major respiratory quinone. To analyze their fatty acid composition, the bacterial cells were cultivated in R2B at 30°C for 7 d, collected, and utilized directly in 10% (v/v) acetyl chloride in methanol at 100°C for 3 h. The resulting fatty acid methyl esters (FAMEs) were extracted with n-hexane thrice. Subsequently, the extracted FAMEs were analyzed using a gas chromatograph (GC) (model GC-1700; Shimadzu Corporation, Kyoto, Japan) equipped with a flame ionization detector and capillary column (BPX70; 0.22 mm [inner diameter] × 50 m, SGE Analytical Science). Helium was employed as the carrier gas. Both the injector and detector were maintained at 260°C. The column temperature was programmed to increase from 160°C to 240°C at increments of 10°C/min and then maintained at 240°C for 7 min [39]. Compounds identified via GC were matched with known standards based on their retention times. The quinones were extracted and analyzed using an ultra-performance liquid chromatography (UPLC) system (Acquity UPLC H-Class system; Waters, Milford, MA) equipped with a photodiode array detector (UPPDA-E; Waters), an Eclipse plus C_18_ column (2.1 × 150 mm; pore size, 1.8 μm; Agilent Technologies, Santa Clara, CA, USA), and MassLynx v4.2 software (Waters), as described previously [40].

### Characterization of rRNA operon numbers and biosynthetic gene clusters (BGCs)

In this study, we also aimed to assess the potential growth rate of the strain by calculating the rRNA operon numbers. Genomic data for our target strain and related species were collected from the EzBioCloud database (https://www.ezbiocloud.net/), and annotation was performed using the DFAST (https://dfast.ddbj.nig.ac.jp/) online server to identify genes, including those encoding the 5S, 16S, and 23S rRNAs. We then estimated the genome size and rRNA operon numbers by calculating the averages weighted by relative abundance in the database (EzBioCloud database) for related phylum species. Data production and visualization were accomplished using GraphPad Prism v9.5.0 software.

To identify and understand the genetic pathways responsible for the production of bioactive compounds, the antiSMASH v7.0.1 bioinformatics tool [41], along with Cluster BLAST, Cluster Pfam analysis, ClusterBlast, ActiveSiteFinder, Pfam-based GO term annotation, SubClusterBlast, RREFinder, and TIGRFam analysis, was used to profile the BGCs in the genome of the ASA1^T^ strain. Furthermore, antiSMASH was linked with the Minimum Information about a Biosynthetic Gene Cluster (MiBIG: v3.0) database (https://mibig.secondarymetabolites.org/), enabling the calculation of cluster sequence similarities through comparisons with experimentally characterized genes used to identify the structure of small molecules [42].

## Results and discussion

### Isolation of the slow-growing strain ASA1^T^ and determining its preferred growth medium

A method for preparing agar media, known as a “PS” medium, involves autoclaving phosphate and agar separately; it was previously proposed to enhance the growth of slow-growing bacteria [16]. This approach is intended to minimize the generation of reactive oxygen species that can inhibit microbial colony formation. In this study, a 100-fold diluted PYG-based PS medium allowed the isolation of strain ASA1^T^, which was related to the rarely cultivated phylum *Verrucomicrobiota*. In a survey of medium preference, strain ASA1^T^ did not produce visible colonies even after 2 weeks of incubation on nutrient-rich media plates (full-strength PYG, LB, NA, and TSA). However, less nutrient-rich media plates (R2A, diluted R2A, and diluted PYG media) elicited visible microbial growth at 8 d of incubation, with full colonies appearing after 14 d of incubation. R2B showed the best growth in liquid culture; it required 7 d for the OD_600_ value (0.3) to increase. Based on the above results, the newly isolated ASA1^T^ strain was considered a slow-growing bacterium that prefers low-nutrient media.

Bacteria possessing multiple rRNA operons typically exhibit faster growth rates due to their enhanced ribosome synthesis capacity [43]. Our investigation into the rRNA operon numbers of closely related *Verrucomicrobiota* species revealed that approximately 90% of these bacteria contained only a single set of rRNA operons (other soil-derived fast-growing bacterial groups including *Bacillus* sp., *Arthrobacter* sp., and *Pseudomonas* sp., have more than 3–4 sets of rRNA operons) (S2 Fig in Appendix S1). This finding correlates with the observed growth characteristics of *Verrucomicrobiota*, resulting in slower growth rates than other bacteria with multiple rRNA operons.

### Phylogenetic and phylogenomic affiliation of strain ASA1^T^

**Fig 1:**
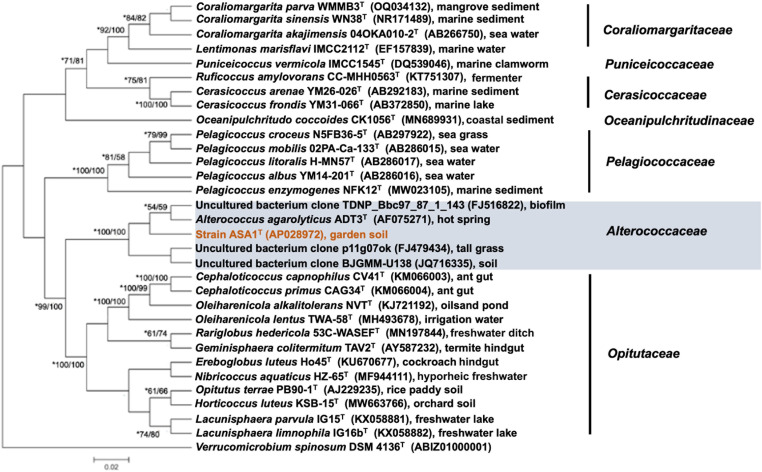
A neighbor-joining tree illustrating the relationship between strain ASA1^T^ and type strains of class *Opitutia* based on their 16S rRNA gene sequences. Bootstrapping was carried out with 1,000 replicates; only values exceeding 50% are displayed. Asterisks (*) denote the distance score in the maximum-likelihood method (S3 Fig in Appendix S1). *Verrucomicrobium spinosum* DSM 4136^T^ served as an outgroup. The bar set at 0.02 signifies the nucleotide substitutions per position.

Based on the 16S rRNA gene sequence comparisons, strain ASA1^T^ exhibited a low sequence identity (<93%) to the closest type strain, *Alterococcus agarolyticus* ADT3^T^ (accession no. NR_036763), of the family *Alterococcaceae* of the class *Opitutia* (phylum *Verrucomicrobiota*). This family was only recently proposed [44] and, at the time of writing, includes only one type strain, *A. agarolyticus*. Consistent with this, ASA1^T^ was attributed to the *Alterococcaceae* cluster in the 16S rRNA gene-based phylogenetic tree (Fig 1, maximum-likelihood tree presented in supplementary S3 Fig in Appendix S1). Its related uncultivated environmental clones have been detected in biofilms (FJ516822), tall grass (FJ479434), and soil (JQ716335); however, their sequence identities are also low (<93%). Collectively, these data suggest that strain ASA1^T^ represents a novel candidate within the family *Alterococcaceae*.

**Fig 2:**
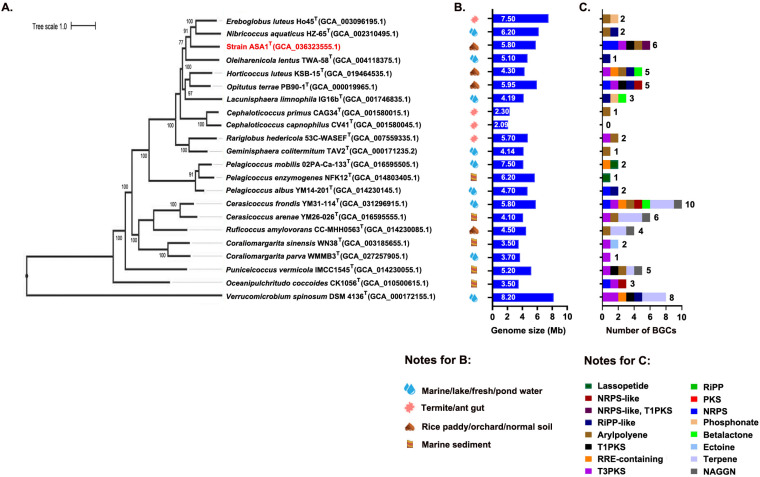
Phylogenomic tree with biosynthetic gene clusters (BGCs) count. (A) A phylogenomic tree coupled with the BGCs count was created using 100 conserved concatenated proteins from bacteria, illustrating the evolutionary relationships between strain ASA1^T^ and the type strains belonging to class *Opitutia*. The root of the tree was established using *V. spinosum* DSM 4136 ^T^ (GCA_000172155.1). Bootstrap values for nodes exceeding 80 are depicted on the tree. The scale bar set at 1.0 represents the amino acid substitutions per position. (B) The isolation source and genomic size of each taxon are also displayed. (C) Bar chart providing an in-depth analysis of the BGCs identified within the genomes of the species in the phylogenomic tree. BGCs were determined using the antiSMASH online tool v7.0.1. The detailed features of the genome and BGCs profiling of the corresponding species are listed in supplementary S1 Table in Appendix S2.

Through genome-wide comparisons, the genome sequence of strain ASA1^T^ revealed low ANI values (<77%) when compared to the closest genomes, type strain genome *Rariglobus hedericola* 53C-WASEF^T^ (MN197844) and MAG VE3 (GCA_014879845.1) (at the time of writing, the genome of *Alterococcus agarolyticus* is not yet available). Thereafter, we generated a phylogenomic tree utilizing the BV-BRC online tool to illustrate the taxonomic relationships of type strains associated with the class *Opitutia* (Fig 2, their BGCs counts are discussed below). This tree was based on the 100 single-copy marker genes of the Bac100 set (Fig 2A) [30]. We also analyzed the phylogenomic placement of strain ASA1^T^ using GTDB-Tk v2.3.2 [45], following the GTDB taxonomy based on 120 bacterial marker genes (S4 Fig in Appendix S1). Two different analyses, via BV-BRC and GTDB-tk, supported the idea that this strain could be a novel member at the genus level.

The AAI values indicated that ASA1^T^ shares <54% AAI with all available *Opitutia* genomes, with the highest similarity observed for HZ-65^T^ (54.0%) and the closest described genus PB90-1^T^ (53.8%) (S1 Table in Appendix S2). As AAI values below 60–65% generally indicate genus-level separation [46,47], these results support the delineation of ASA1^T^ as a novel genus within the class *Opitutia*. ASA1^T^ represents the only available genome from a cultivated strain within the family *Alterococcaceae*, highlighting the need for further cultivation efforts and genome sequencing of related taxa to better resolve phylogenomic relationships within *Opitutia*.

### Morphological and biochemical characterization of strain ASA1^T^

**Fig 3:**
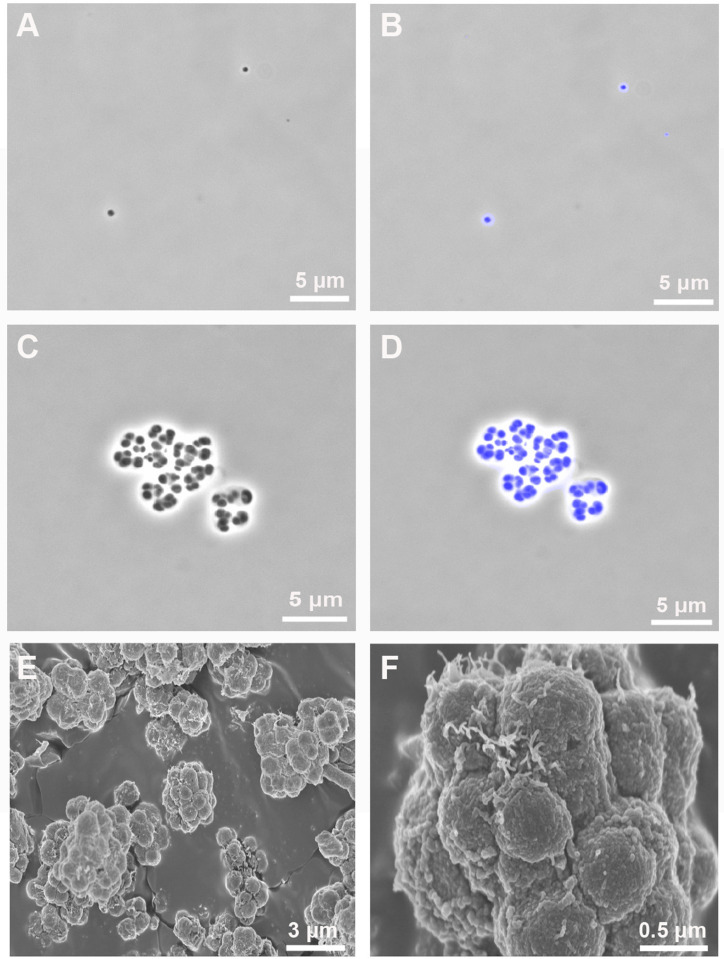
Phase-contrast, fluorescence (DAPI-stained), and scanning electron microscopy (SEM) images of strain ASA1^T^. Images (A–D) were observed after 7 d of incubation on R2B medium at 30°C. The image in (A) shows small coccus- and oval-shaped cells during the exponential phase (7 d), while that in (C) demonstrates cluster-like cell aggregation under the same conditions. (B) and (D) are overlays of (A) and (C) with DAPI, respectively. (E) and (F) show SEM images of cluster-like aggregates formed after a longer incubation period (two weeks). Scale bars are 5 μm for images (A–D), 3 μm for image (E), and 0.5 μm for image (F).

Colonies of strain ASA1^T^, cultivated on R2A plates at 30°C for 8 d, displayed a whitish-yellow color. Under light microscopy, cells were coccus- or oval-shaped, and cell sizes were 0.45 × 0.45–0.60 µm (corresponding to cell volumes ranging from 0.05 to 0.07 μm^3^, Figs 3A–3B). These small cells displayed typical characteristics of ultramicrobacteria, which are defined as bacteria with cell volumes of less than 0.1 μm^3^ [11]. Anaerobic verrucomicrobacterial ultramicrobacteria have already been reported [12]; however, to our best knowledge, strain ASA1^T^ is the first cultivated representative of an aerobic one. This strain also forms >10-µm-sized cluster-like aggregates (Figs 3C–3F). A similar cell morphology has been observed in a certain *Acidobacteriota* member [48]. The acidobacterial strain SBC82^T^ forms large aggregates and exhibits saccular chambers and cellulose-made extracellular structures. Moreover, X-ray microanalysis revealed the presence of silicon in their chamber walls, enhancing mechanical rigidity [49,50]. As to whether ASA1^T^ aggregates function similarly to those of strain SBC82^T^, additional data (e.g., analysis of the cell structure and composition) are needed.

Strain ASA1^T^ exhibited the following characteristics: Gram-negative, motile, aerobic, negative to catalase, KOH, and oxidase. The KOH solubility test for strain ASA1^T^ showed a negative reaction (no viscosity), typically indicating a Gram-positive profile. However, Gram staining consistently showed pink/red cells, suggesting a Gram-negative identity. Previous studies have revealed discrepancies between the KOH test and Gram staining, with certain bacterial strains producing inconsistent results due to differences in cell envelope structure [51, 52]. Furthermore, the majority of *Verrucomicrobiota* are morphologically characterized as Gram-negative [53,54], supporting this interpretation. Regardless of the KOH-negative result, the Gram-negative identification of ASA1^T^ was accepted based on consistent Gram staining. The absence of catalase activity suggests that using PS medium, which reduces the generation of hydrogen peroxide, likely contributed to the isolation of this strain [55]. The motility of strain ASA1^T^ was confirmed by growth extending beyond the inoculation line on a soft agar medium, as well as through direct observation using optical microscopy. The growth temperature of strain ASA1^T^ ranged from 20–40°C, with optimal growth at 30°C. The pH range for its growth was 7.0–11.0, with the highest growth observed at pH 8.4. The soil from which the ASA1^T^ strain was obtained had a pH of around 8. Additionally, bacterial growth was observed in R2B medium containing 0–3.0% NaCl, with maximum growth at 0% NaCl and no growth above 3.0% NaCl. In the API 20NE and API ZYM tests, alkaline phosphatase and esterase (C4) were positive, and esterase lipase (C8), napthol-AS-BI-phosphohydrolase, α-galactosidase, and β-galactosidase were weakly positive, whereas all other tests were negative. The other phenotypic features of strain ASA1^T^ and the other phylogenetically related type strains are presented in Table 1.

**Table 1:** Differentiation between strain ASA1^T^ and other closely related representatives of *Opitutales.*

Features	1	2	3	4	5	6	7	8	9	10
Isolation source	Garden soil	Hot springs	Orchard soil	Rice paddy soil	Termite gut	Sea polychaete	Freshwater	Oil sands tailing ponds	Hyporheic freshwater	Mangrove sediment
DNA G + C content (mol%)	65.6	65.8	64.3	74.0	60.5	52.1	60.6	66.1	62.2	56.0
Cell shape	Coccus or oval	Coccus or spherical	Coccus or short rod	Coccus	Diplococcus	Coccus	Coccus	Coccus or diplococcus	Coccus	Coccus
Cell diameter (μm)	Coccus or oval (0.4–0.6) and aggregate (>10 µm)	0.8–0.9	0.4–0.7	0.4–0.6	0.5–0.6	0.6–1.0	0.35	0.5–1.0	0.4–0.5	ND
Colony color	Whitish yellow	White	Yellow	Colorless	Colorless	Pale reddish	Colorless	White	Yellow	ND
Metabolism	Obligate aerobe	Facultative anaerobe and aerobe	Obligate aerobe	Obligate anaerobe	Obligate aerobe	Facultative anaerobe	Obligate aerobe	Obligate aerobe	Obligate aerobe	Obligate aerobe
Motility	+	+	–	+	–	–	+	–	–	–
Catalase	–	+	+	–	–	–	–	+	–	+
Oxidase	–	+	–	–	–	–	–	–	–	+
Temp. (°C)	20–40 (30)	40–56	15–37 (25)	10–37	15–35 (30)	10–40 (30–35)	6–30	15–40 (25–37)	15–37 (25–30)	15–40 (30)
Growth pH	7.0–11.0 (8.4)	7.0–8.5 (8.0)	5.0–11.0 (6.0)	5.5–9.0 (7.5–8.0)	5.5–7.5 (7.0)	5.5–9.5 (7.0–7.5)	ND	5.5–11.0 (6.0–8.0)	5.5–9.0 (7.0)	ND
NaCl tolerance (%, w/v)	0–3.0 (0)	1.0–3.5 (2.0–2.5)	0–3.0 (0)	3.0	1.5	1.0–10.0 (2.5–3.0)	0–0.7	0–3.0	0–0.5 (0)	ND
**Substrates**										
Arabinose	–	–	+	+	–	W	ND	W	+	+
Cellobiose	–	+	+	+	+	+	ND	–	+	ND
Fructose	–	ND	–	+	–	+	+	+	+	ND
Glucose	+	+	+	+	+	+	W	+	+	+
Galactose	–	+	–	+	+	+	ND	+	+	ND
Maltose	+	ND	–	+	+	+	ND	–	ND	ND
Mannitol	–	–	ND	+	–	+	ND	+	–	w
Mannose	–	W	–	+	–	+	ND	–	+	ND
Ribose	–	ND	ND	–	–	ND	ND	ND	ND	ND
Starch	–	ND	–	+	+	ND	ND	–	–	–
Sorbitol	–	–	ND	ND	–	+	ND	+	ND	ND
Sucrose	+	+	–	+	–	+	ND	–	–	ND
Xylose	–	+	+	–	–	ND	ND	+	ND	ND
Major fatty acids (%)	anteiso-C_15:0_ (53.0), C_16:0_ (15.0)	anteiso-C_15:0_ (51.5)	iso-C_15:0_ (34.9), anteiso-C_15:0_ (29.0)	ND	ND	anteiso-C_15:0_ (30.9), C_18:0_ (24.7), C_16:0_ (7.9), and C_17:0_ (7.0)	anteiso-C_15:0_ (31.1), C_16:0_ (26.7), C_16:1_ω5c (16.9), iso-C_14:0_ (8.3), and C_14:0_ (6.0)	anteiso-C_15:0_ (28.4), iso-C_15:0_ (17.1)	iso-C_14:0_ (19.9), anteiso-C_15:0_ (19.3), C_16:0_ (16.8), and iso-C_16:0_ (14.5)	iso-C_14:0_ (23.1) and C_18:1_ *ω*9*c* (19.0)
Respiratory quinones	MK-7	ND	MK-7 and MK-6	ND	ND	MK-7	MK-7	MK-7 and MK-6	MK-7	MK-7

Strains: 1. ASA1^T^; 2. *Alterococcus agarolyticus* ADT3^T^ [56,57]; 3. *Horticoccus luteus* KSB-15^T^ [58]; 4. *Opitutus terrae* PB90-1^T^ [59]; 5. *Geminisphaera colitermitum* TAV2^T^ [15,60]; 6. *Puniceicoccus vermicola* IMCC1545^T^ [61]; 7. *Rariglobus hedericola* 53C-WASEF^T^ [62]; 8. *Oleiharenicola alkalitolerans* NVT^T^ [63]; 9. *Nibricoccus aquaticus* HZ-65^T^ [64]; 10. *Coraliomargarita parva* WMMB3^T^ [44]; (+) present or utilized; (-) absent or not utilized; (w) weakly positive; (ND) not determined. Values in parentheses indicate the optimal conditions.

In summary, the combination of ultramicrobacterial morphology, clustered cells formation, and temperature growth preference contributes to the delineation of strain ASA1^T^ from currently described members of *Opitutales*. These physiological and morphological traits, along with phylogenetic and genomic evidence, substantiate its classification as a representative of a novel genus within the phylum *Verrucomicrobiota*.

### Habitat distribution and preferences of strain ASA1^T^

An IMNGS analysis was conducted to explore the potential distribution and habitat preferences of strain ASA1^T^ and its related *Verrucomicrobiota* members. The IMNGS search involved comparing the genetic information of ASA1^T^ to 16S rRNA gene amplicon datasets. At a similarity threshold of >97%, ASA1^T^ was found to match 229 datasets, with most of its source environments being the soil (88 datasets), aquatic sources (32), and activated sludge (21). To delve deeper into its habitat preferences, additional searches were conducted using ProkAtlas. The ProkAtlas results showed that strain ASA1^T^ relatives exhibited potential preferences for various environments (S5 Fig in Appendix S1). At a 97% threshold, the top habitats included freshwater (20.82%), peat (15.10%), rhizosphere (11.12%), soil (10.88%), aquatic (9.62%), lake water (8.13%), groundwater (7.12%), wetland (6.69%), freshwater sediment (6.41%), and marine (4.07%) environments. These results suggest that relatives of ASA1^T^ are distributed in various environments.

### Chemotaxonomic profiling analysis of ASA1^T^

The dominant cellular fatty acids of ASA1^T^ were anteiso-C_15:0_ (53%) and C_16:0_ (15%). Other fatty acids, such as C_16:1_ ω7c (9.0%), C_18:1_ (7.0%) (containing two isomers), anteiso-C_17:0_ (5.0%), C_17:0_ (4.0%), C_15:0_ (2.0%), C_12:0_ 3-OH (1.0%), C_13:0_ 3-OH (1.0%), and iso-C_16:0_ (1.0%), were also detected. A previous study highlighted the pivotal roles of anteiso-C_15:0_ fatty acids in bacterial adaptation to environmental stresses and the modulation of membrane fluidity in response to temperature fluctuations [65,66]. While the precise physiological impact of anteiso-C_15:0_ on the membrane fluidity response of ASA1^T^ remains unclear, its abundant presence may have implications for environmental stress mechanisms. Its dominant isoprenoid quinone was menaquinone-7 (MK-7), consistent with the other representative strains of the order *Opitutales* (Table 1).

### Secondary metabolite biosynthetic potential of *Opitutales* members

Recent advancements in microbial genomics have unveiled novel BGCs from challenging-to-culture bacteria such as *Acidobacteriota*, *Verrucomicrobiota*, and *Gemmatimonadota* [67]. In this study, we preliminarily identified potential BGCs responsible for producing novel secondary metabolites in the ASA1^T^ strain and related species. Using antiSMASH v7.0.1, we predicted and analyzed BGCs profiles across 22 genomes, revealing a consistently lower BGCs count per genome (1–10 clusters) (Fig 2C). No direct correlation was found regarding the relationship between BGCs content and genome size (Fig 2B). Notably, soil-derived *Opitutales* strains had clusters relevant for biotechnological applications, including non-ribosomal peptide synthetase (NRPS), type I polyketide synthase (T1PKS), type III polyketide synthase (T3PKS), ribosomally synthesized and post-translationally modified peptide (RiPP), and NRPS-like clusters [68]. In the ASA1^T^ genome, we predicted six notable BGCs regions comprising five types of BGCs, including NRPS, T3PKS, NRPS-like, arylpolyene, and T1PKS clusters. These BGCs encompassed core biosynthetic, regulatory, and other genes (S6 Fig in Appendix S1). Moreover, these BGCs also exhibited low similarity scores (<1%) to known clusters, suggesting they may encode novel secondary metabolites for future studies.

### Proposal for a new taxon, *Congregicoccus parvus* gen. nov., sp. nov

The newly isolated strain ASA1^T^ exhibited low 16S rRNA gene sequence identities (<93%), ANI values (<77%), and AAI values (<54%) compared to type strains within the class *Opitutia* of the phylum *Verrucomicrobiota*. Distinct phenotypic and physiological variations were also observed when comparing ASA1^T^ to *A. agarolyticus* ADT3^T^, the only described species of *Alterococcaceae*. Unlike *A. agarolyticus*, which thrives at higher temperatures (40–56°C) and was isolated from a hot spring, ASA1^T^ was recovered from garden soil and prefers a lower temperature range (20–40°C, optimum at 30°C). Additionally, ASA1^T^ exhibits an ultramicrobacterial morphology (0.4–0.6 µm) with cluster-like aggregation (>10 µm), whereas *A. agarolyticus* cells are larger (0.8–0.9 µm) and non-aggregating. ASA1^T^ is aerobic, catalase and oxidase negative, whereas *A. agarolyticus* is facultatively anaerobic, catalase and oxidase positive. The high cellular anteiso-C_15:0_ (53%) content of ASA1^T^ was shared with that of *A. agarolyticus*, but was different from those of the members belonging to other families. Based on its phylogenetic, phenotypic, physiological, and chemotaxonomic characteristics, we propose *Congregicoccus parvus* gen. nov., sp. nov. for this strain.

### Description of *Congregicoccus* gen. nov

*Congregicoccus* (Con.gre.gi.coc’cus. L. masc. adj. *congregus*, united in flocks; N.L. masc. n. *coccus* [from Gr. masc. n. *kokkos*, grain, seed], coccus; N.L. masc. n. *Congregicoccus*, a coccus that grows in flocks).

Cells are small, form cluster-like aggregates, and grow chemoorganotrophically and aerobically. These are Gram-negative, motile, and non-spore-forming. The major fatty acids are anteiso-C_15:0_ and C_16:0_; the major isoprenoid quinone is menaquinone-7 (MK-7).

This genus is classified under the family *Alterococcaceae*. The type species is *Congregicoccus parvus*.

### Description of *Congregicoccus parvus* sp. nov

*Congregicoccus parvus* (par’vus. L. masc. adj. *parvus*, small, referring to small cells of the type strain).

Colonies grown on R2A plates are whitish-yellow, smooth, < 1.0 mm in diameter, and slightly raised. After extended incubation, the colony color changed slightly to yellow. Cells were coccus- or oval-shaped, with sizes ranging from 0.45 × 0.45–0.60 µm (corresponding cell volumes ranging from 0.05–0.07 μm^3^). Cells also formed >10-µm-sized cluster-like aggregates. The temperature range for growth on R2A medium is 20–40°C (optimal at 30°C). The pH range for growth is 7.0–11.0, with the optimum at 8.4 (potential changes in pH during incubation can affect colony growth). The NaCl concentration for growth ranged from 0% to 3.0%, with maximum growth at 0% NaCl and no growth above 3.0% NaCl. Cells lack the presence of catalase, KOH, and oxidase activities. Positive enzymatic activities were observed for alkaline phosphatase and esterase (C4) reactions, with weakly positive reactions noted for esterase lipase (C8), napthol-AS-BI-phosphohydrolase, α-galactosidase, and β-galactosidase; all other tests yielded negative results. Substrates utilized as energy sources include D-glucose, sucrose, and D-maltose.

The strain ASA1^T^ (= JCM 36569^T^ = NCIMB 15508^T^) was isolated from the garden soil at the AIST Hokkaido Center, Sapporo, Hokkaido, Japan. Its DNA G + C content was approximately 66%. Its genome was approximately 5.8 Mbp. The ASA1^T^ genome sequence was deposited in the DDBJ/ENA/GenBank database under accession number AP028972.1 (BioProject/BioSample PRJDB16742/SAMD00647589; DDBJ Sequence Read Archive DRA017385).

## Supporting information

Appendix S1 S1 Fig. Schematic representation of the circular genome map of the ASA1^T^ strain generated using the CGView server online tool (http://cgview.ca/). Circle 1 (outermost) displays the coding sequence. Circles 2 and 3 display the AT content plot. Circles 4 and 5 (innermost) display the GC content and GC skew, respectively. S2 Fig. Analysis of 19 genomes from the phylum *Verrucomicrobiota* conducted using EzBioCloud (https://www.ezbiocloud.net/) revealed a correlation between rRNA operon numbers and incubation times for optimal growth. Approximately 90% of species exhibited single rRNA operons, which correlates with the prolonged incubation times required for optimal growth. The estimation of the operon numbers and incubation times was based on data provided in supplementary S1 Table in Appendix S2, which includes the incubation time for each isolate, with references. **S3 Fig. Maximum-likelihood tree was constructed to illustrate the relationship between strain ASA1^T^ and type strains of the class *Opitutia*.** Bootstrapping was carried out with 1,000 replicates; only values exceeding 50% are displayed. The scale bar represents a sequence difference of 0.02. *Verrucomicrobium spinosum* DSM 4136^T^ was employed as the outgroup. **S4 Fig. Phylogenomic tree generated using 120 concatenated bacterial conserved proteins to elucidate the relationships among genomes and MAGs associated with the class *Opitutia*.** Type strains are highlighted in red boxes. Clades lacking cultivated representatives were condensed for clarity. **S5 Fig. Habitat preference prediction of strain ASA1^T^ and its related *Verrucomicrobiota* members based on ProkAtlas.** **S6 Fig. Graphical representation of six notable secondary metabolite gene cluster regions identified within the ASA1 genome (generated from antiSMASH v7.0.1).** The cluster regions are labeled A, B, C, D, E, and F, and the positions of core biosynthetic genes, regulatory elements, and other genes associated with secondary metabolite production are highlighted. Additionally, the locations of additional biosynthetic genes within each cluster, which are potentially responsible for the synthesis of active bioactive compounds, are indicated. **S1 Method. Scanning electron microscope (SEM) observation.**
(DOCX)

Appendix S2 S1 Table. List of genomes used in this study to detect rRNA operons, AAI values, and secondary metabolite gene clusters. (XLSX)
