# Supplementary material for: Characterization of Congregicoccus parvus gen. nov., sp. nov., a novel slow-growing ultramicrobacterium of the phylum Verrucomicrobiota
Source: PLoS One. 2025 Jun 25;20(6):e0326734. doi: 10.1371/journal.pone.0326734 (PMC12193912; doi:10.1371/journal.pone.0326734)
Supplement: Appendix S1 — Approximately 90% of species exhibited single rRNA operons, which correlates with the prolonged incubation times required for optimal growth. The estimation of the operon numbers and incubation times was based on data provided in supplementary S1 Table in Appendix S2, which includes the incubation time for each isolate, with references. S3 Fig. Maximum-likelihood tree was constructed to illustrate the relationship between strain ASA1T and type strains of the class Opitutia. Bootstrapping was carried out with 1,000 replicates; only values exceeding 50% are displayed. The scale bar represents a sequence difference of 0.02. Verrucomicrobium spinosum DSM 4136T was employed as the outgroup. S4 Fig. Phylogenomic tree generated using 120 concatenated bacterial conserved proteins to elucidate the relationships among genomes and MAGs associated with the class Opitutia. Type strains are highlighted in red boxes. Clades lacking cultivated representatives were condensed for clarity. S5 Fig. Habitat preference prediction of strain ASA1T and its related Verrucomicrobiota members based on ProkAtlas. S6 Fig. Graphical representation of six notable secondary metabolite gene cluster regions identified within the ASA1 genome (generated from antiSMASH v7.0.1). The cluster regions are labeled A, B, C, D, E, and F, and the positions of core biosynthetic genes, regulatory elements, and other genes associated with secondary metabolite production are highlighted. Additionally, the locations of additional biosynthetic genes within each cluster, which are potentially responsible for the synthesis of active bioactive compounds, are indicated. S1 Method. Scanning electron microscope (SEM) observation. (DOCX) [file pone.0326734.s001.docx]

**Characterization of *Congregicoccus parvus* gen. nov., sp. nov., a novel slow-growing ultramicrobacterium of the phylum *Verrucomicrobiota***

-Supplementary materials-

Md. Samiul Islam^1,2^, Kyosuke Yamamoto^2^, Naoki Morita^1,2^, Isao Yumoto^1,2^, Wataru Kitagawa^1,2^, Souichiro Kato^1,2^, Ryosuke Nakai^2*^, Kensuke Igarashi^2*^

-Affiliation-

^1^ Graduate School of Agriculture, Hokkaido University, Sapporo, Hokkaido, Japan

^2^ Bioproduction Research Institute, National Institute of Advanced Industrial Science and Technology (AIST), Sapporo, Hokkaido, Japan


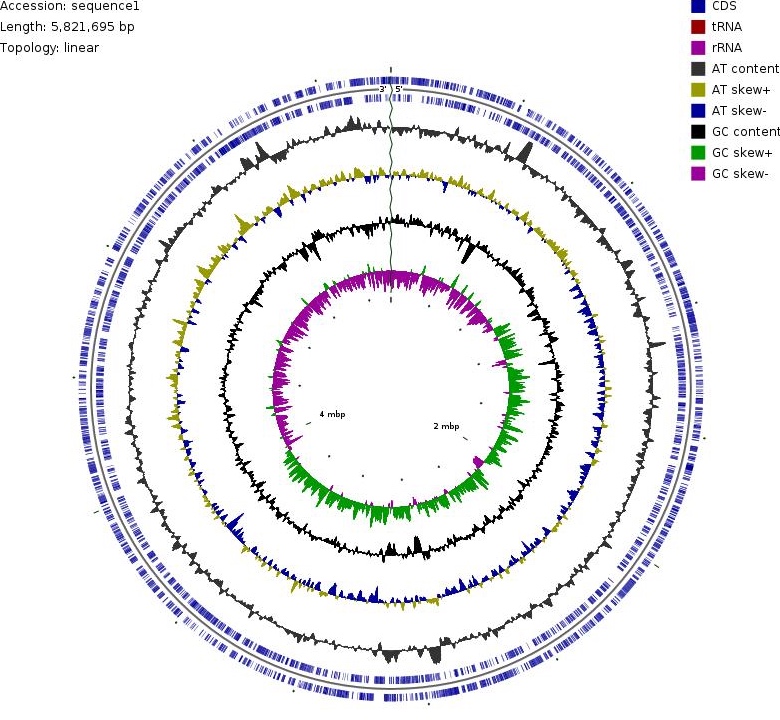


**S1 Fig.** **Schematic representation of the circular genome map of the ASA1^T^ strain generated using the CGView server online tool (**[**http://cgview.ca/**](http://cgview.ca/)**).** Circle 1 (outermost) displays the coding sequence. Circles 2 and 3 display the AT content plot. Circles 4 and 5 (innermost) display the GC content and GC skew, respectively.





**S2 Fig.** **Analysis of 19 genomes from the phylum *Verrucomicrobiota* conducted using EzBioCloud (https://www.ezbiocloud.net/) revealed a correlation between rRNA operon numbers and incubation times for optimal growth.** Approximately 90% of species exhibited single rRNA operons, which correlates with the prolonged incubation times required for optimal growth. The estimation of the operon numbers and incubation times was based on data provided in supplementary S1 Table in Appendix S2, which includes the incubation time for each isolate, with references.


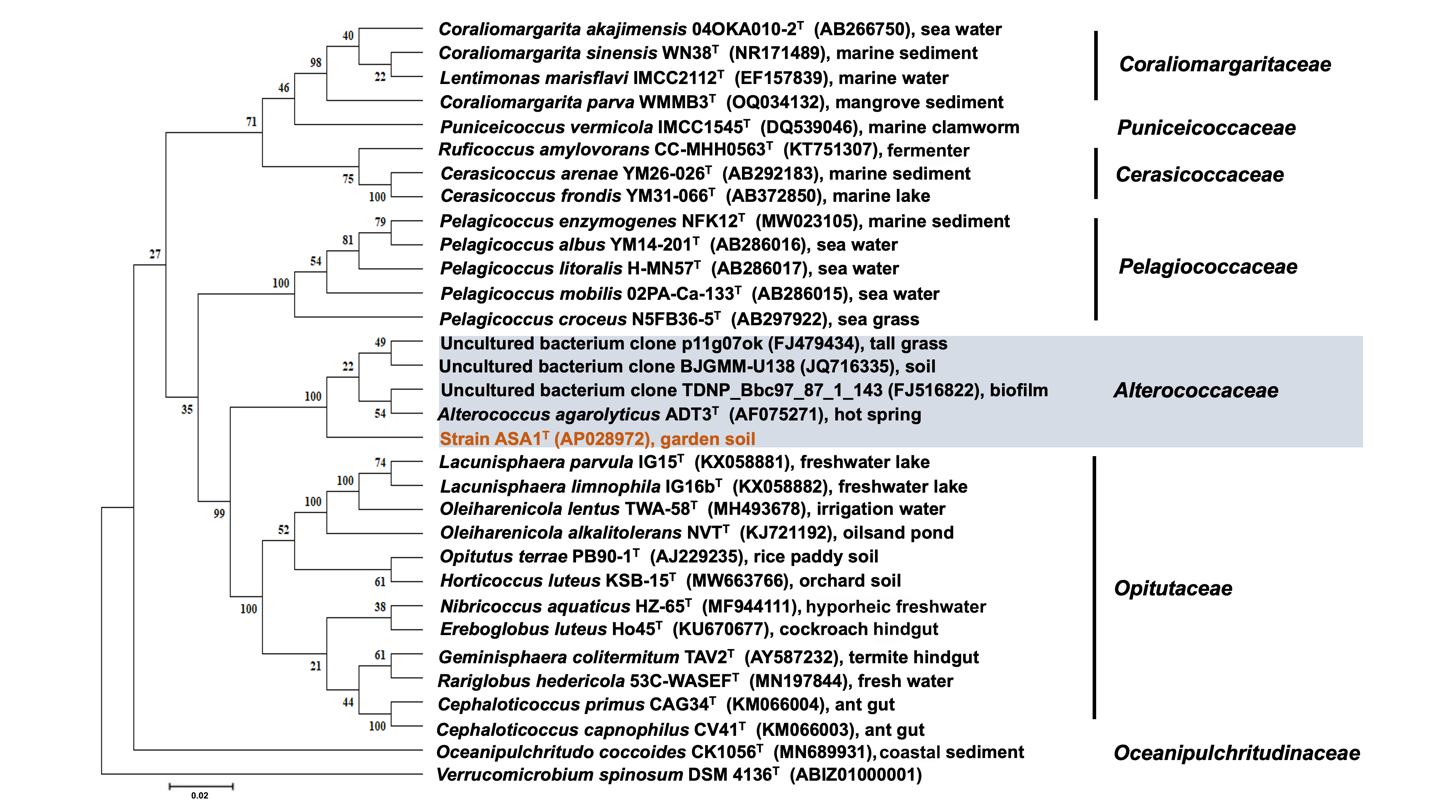


**S3 Fig.** **Maximum-likelihood tree was constructed to illustrate the relationship between strain ASA1^T^ and type strains of the class *Opitutia*.** Bootstrapping was carried out with 1,000 replicates; only values exceeding 50% are displayed. The scale bar represents a sequence difference of 0.02. *Verrucomicrobium spinosum* DSM 4136^T^ was employed as the outgroup.


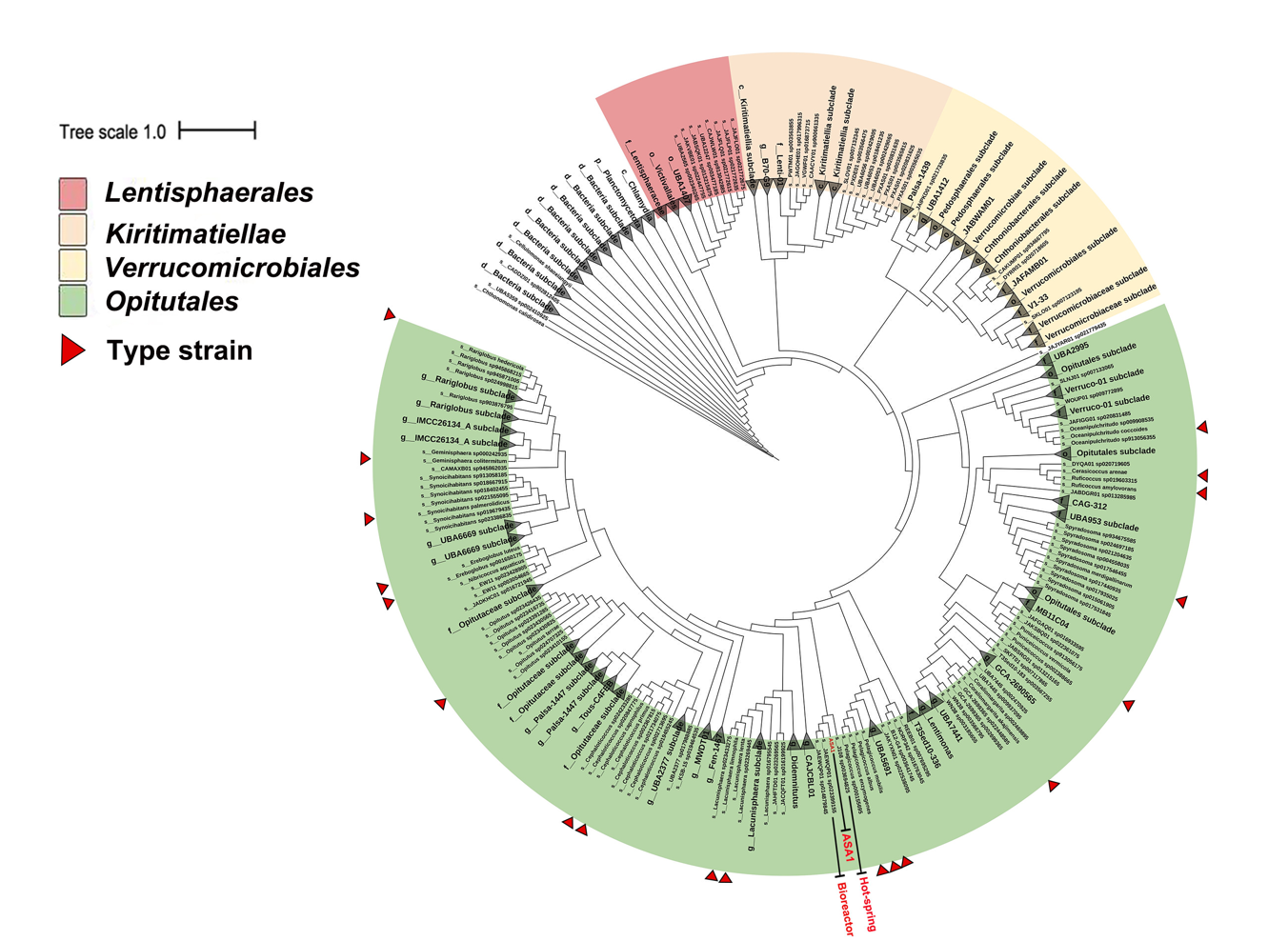


**S4 Fig. Phylogenomic tree generated using 120 concatenated bacterial conserved proteins to elucidate the relationships among genomes and MAGs associated with the class *Opitutia*.** Type strains are highlighted in red boxes. Clades lacking cultivated representatives were condensed for clarity.





**S5 Fig. Habitat preference prediction of strain ASA1ᵀ and its related *Verrucomicrobiota* members based on ProkAtlas.**


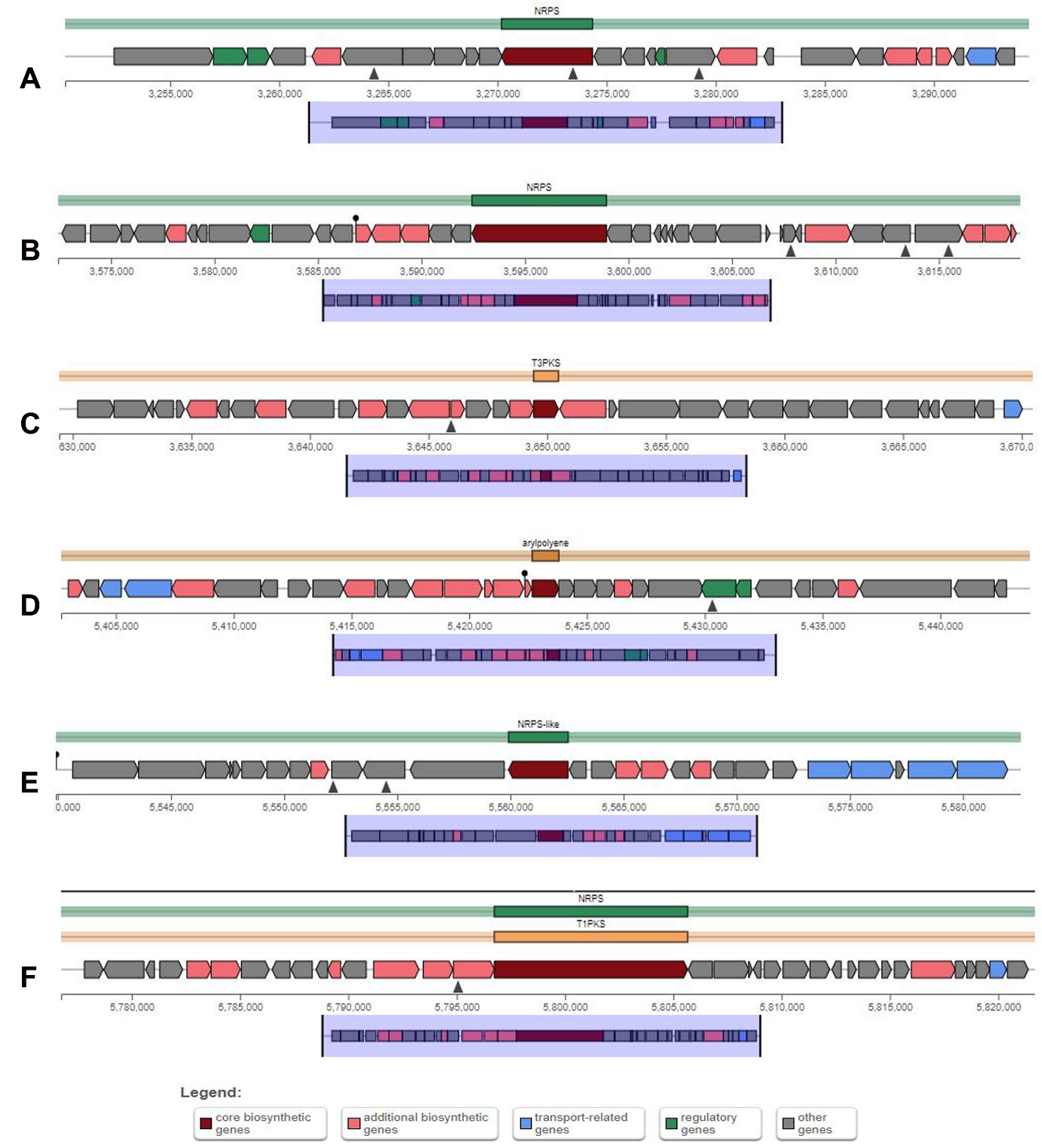


**S6 Fig. Graphical representation of six notable secondary metabolite gene cluster regions identified within the ASA1 genome (generated from antiSMASH ver. 7.0.1).** The cluster regions are labeled A, B, C, D, E, and F, and the positions of core biosynthetic genes, regulatory elements, and other genes associated with secondary metabolite production are highlighted. Additionally, the locations of additional biosynthetic genes within each cluster, which are potentially responsible for the synthesis of active bioactive compounds, are indicated.

**S1 Method. Scanning electron microscope (SEM) observation**

For SEM, ASA1^T^ cell samples were fixed with 2% paraformaldehyde and 2% glutaraldehyde in 0.1 M sodium cacodylate buffer (pH 7.4) at 4 °C for 3 d. Then, the sample was additionally fixed with 1% tannic acid in cacodylate buffer at 4 °C for 2 h. After fixation, the sample was washed four times with cacodylate buffer for 30 min each, followed by post-fixation with 2% osmium tetroxide in cacodylate buffer at 4 °C for 2 h. The sample was then dehydrated through a graded ethanol series, keeping it in 50% and 70% ethanol for 30 min each at 4 °C, in 90% ethanol for 30 min at room temperature, followed by four washes in 100% ethanol at room temperature, each lasting for at least 30 min. The dehydrated sample was gradually subjected to a 1:1 mixture of ethanol and *tert*-butyl alcohol for 1 h, then 100% *tert*-butyl alcohol for 1 h, frozen at 4 °C, and subjected to vacuum freeze drying. After drying, the samples were coated with a 30 nm layer of osmium using an osmium plasma coater (NL-OPC80A; Nippon Laser & Electronics Laboratory, Nagoya, Japan). Coated samples were observed using a SEM (JSM-7500F; JEOL Ltd., Tokyo, Japan) at an acceleration voltage of 3.0 kV, with digital images captured directly onto a computer and archived onto a compact disc.
